# Using causal methods to map symptoms to brain circuits in neurodevelopment disorders: moving from identifying correlates to developing treatments

**DOI:** 10.1186/s11689-022-09433-1

**Published:** 2022-03-12

**Authors:** Alexander Li Cohen

**Affiliations:** 1 Department of Neurology, Boston Children’s Hospital, Harvard Medical School, 300 Longwood Avenue, Boston, MA 02115 USA; 2 Computational Radiology Laboratory, Department of Radiology, Boston Children’s Hospital, Harvard Medical School, Boston, MA USA; 3 Laboratory for Brain Network Imaging and Modulation, Center for Brain Circuit Therapeutics, Department of Neurology, Brigham and Women’s Hospital, Harvard Medical School, Boston, MA USA

**Keywords:** Translational neuroimaging, Transcranial magnetic Stimulation, Transcranial direct current stimulation, Real-time fMRI neurofeedback, Transdiagnostic symptoms, Neurodevelopmental disorders

## Abstract

A wide variety of model systems and experimental techniques can provide insight into the structure and function of the human brain in typical development and in neurodevelopmental disorders. Unfortunately, this work, whether based on manipulation of animal models or observational and correlational methods in humans, has a high attrition rate in translating scientific discovery into practicable treatments and therapies for neurodevelopmental disorders.

With new computational and neuromodulatory approaches to interrogating brain networks, opportunities exist for “bedside-to bedside-translation” with a potentially shorter path to therapeutic options. Specifically, methods like lesion network mapping can identify brain networks involved in the generation of complex symptomatology, both from acute onset lesion-related symptoms and from focal developmental anomalies. Traditional neuroimaging can examine the generalizability of these findings to idiopathic populations, while non-invasive neuromodulation techniques such as transcranial magnetic stimulation provide the ability to do targeted activation or inhibition of these specific brain regions and networks. In parallel, real-time functional MRI neurofeedback also allow for endogenous neuromodulation of specific targets that may be out of reach for transcranial exogenous methods.

Discovery of novel neuroanatomical circuits for transdiagnostic symptoms and neuroimaging-based endophenotypes may now be feasible for neurodevelopmental disorders using data from cohorts with focal brain anomalies. These novel circuits, after validation in large-scale highly characterized research cohorts and tested prospectively using noninvasive neuromodulation and neurofeedback techniques, may represent a new pathway for symptom-based targeted therapy.

## Background

At the intersection of computational neuroscience and developmental cognitive neuroscience are attempts to identify and characterize the brain structures, networks, and processes underlying the development of human behavior. In support of this goal, a variety of techniques have been used including analysis of genetically consistent clinical cohorts and knockout animal models, single cell recording during behavior in awake and behaving animals, and non-invasive neurophysiologic and neuroimaging methods in infants, children, and adolescents [1]. Because there are also so many levels of investigation, e.g., single gene expression, the activity of particular cell types, the synaptic interaction between neurons in the brain, the study of brain regions and the networks between them, as well as human and animal behavior, there are many paths to understand differences and alterations pertinent to human behavior, intelligence, and neurodevelopmental disorders [2].

Nonetheless, translation from discoveries found at the single gene level or from animal models does not always translate into human therapeutics. This is often attributed to the complexity of interactions and emergent properties that connect gene expression to human behavior [3–5]. However, a shorter logical “leap” is possible by connecting alterations at the whole brain or brain network level with differences in behavior or changes in human development. As such, the use of neurophysiologic and neuroimaging methods to identify correlational “endophenotypes” for particular diagnoses and phenotypes is a growing field of its own [6–13]. A subset of biomarkers in general, endophenotypes are quantitative biological traits that reflect the function of a discrete biological system, correlate with disease severity or susceptibility, and are reasonably heritable. As such, they are considered more closely related to the root cause of a disease than the behavioral/clinical phenotype itself [14].

### The challenge of diagnostic and methodologic heterogeneity in correlational studies of neurodevelopmental disorders

Extending beyond the specific aim of identifying diagnostic endophenotypes for disorders, the bulk of neuroimaging in neurodevelopmental disorders has still focused on identifying *any* descriptive imaging-phenotype correlations by comparing affected cohorts [15–17], similar to the identification of genotype-phenotype correlations in genome-wide association studies (GWAS). For instance, the Simons Foundation Powering Autism Research (SPARK) study maintains a growing list of single genes and copy number variants (now at 167 single genes, 43 copy number variants, and 5 chromosomal variants) that are related to autism, many of which have specific genotype-phenotype patterns [18].

Over the past several years, however, it has become apparent that linking neurophysiological or neuroimaging findings to external behavioral phenotypes of a particular neurodevelopmental disorder is a significant challenge. This may be primarily due to lack of reproducibility [19] across studies using the typical sample size (N ~ 20–30) available to individual lab groups [20, 21], but may also be due to multiple sources of heterogeneity across conditions and methods. This has highlighted a need to (1) improve behavioral characterization and differentiation of clinical populations, e.g., “deep phenotyping” [8], (2) identify and reduce meaningful variation due to methodological choices [22], and (3) increase sample sizes to find reproducible and generalizable results through collaboration and consortia efforts [20]. Multiple efforts have already begun to fill each of these gaps, including the Adolescent Brain Cognitive Development (ABCD) project that will longitudinally study over 11,000 children as they progress through adolescence with behavioral, neurophysiological, and neuroimaging-based assessment [23], and the Brain Imaging Data Structure (BIDS) initiative, which seeks to develop an ecosystem of interoperable data and analysis pipelines for studying the brain [24, 25]. Multiple groups are also advancing consistent processing and statistical protocols to generate more reproducible results in neuroimaging and in neurophysiology [26–28].

However, even with the advancements noted above, this still represents a data-driven observational approach to identify statistical correlations and associations. This leads to an often-implicit assumption that with enough data, diagnosis-level biomarkers will emerge. This has been shown to be possible, but the findings that emerge from this type of analysis often have small effect sizes and may miss larger effects in specific subgroups due to group heterogeneity [29]. More importantly, these results are still correlational in nature—without perturbation or modulation, it is difficult to differentiate between findings that (1) represent endophenotypes or proximal causes for a behavioral phenotype, (2) are present because they are attempts to compensate for a behavioral phenotype, or (3) are present because they are downstream effects/biomarkers of some other cause.

A complementary strategy, however, may be to discard etiological or categorical diagnosis labels altogether, and focus purely on identifying the neuroanatomical basis for quantifiable, unidimensional symptoms or aspects of human behavior [30, 31]. Specifically, (1) focusing on individual symptoms across clinical cohorts, particularly those with focal brain injury, atrophy, or other focal brain alterations may provide region- or circuit-based hypotheses that are more likely to represent endophenotypes than observational/diagnosis association-based approaches, (2) traditional neuroimaging study of patients with neurodevelopmental disorders or analysis of large-scale highly characterized cohorts can then be performed in a more hypothesis-driven fashion, (3) if consistent, non-invasive neuromodulatory techniques can test whether these brain circuits can be easily modulated, and (4) if so, this pathway leads directly to clinical trials that test whether non-invasive modulation of these circuits results in measurable symptomatic changes at the group- and individual-level. It is my thesis that treatment targets generated in this fashion are significantly more likely to be effective than those generated from diagnosis-association approaches alone.

## A “bedside-to-bedside” pathway to identify treatment targets for individual symptoms in neurodevelopmental disorders

### Understanding the generation of specific symptoms may be a more direct approach to clinical utility

There is reason to believe that identifying the *specific* neuroanatomical/neurofunctional bases of *specific* unidimensional symptoms will enable the development of targeted therapies in a more rapid fashion than genetically informed drug targets for symptoms in autism spectrum disorder (ASD) and other neurodevelopmental disorders. For instance, patients with Parkinson’s disease have a mix of symptoms and co-morbidities that is nearly as heterogeneous as that in individuals with ASD: some Parkinson’s patients have a tremor, others have difficulty with gait, and yet others have depression or sleep disturbances [32]. These symptoms localize to various brain regions, and respond to different types of medication as well as to stimulation of different brain locations [33, 34]. Conversely, patients with different diseases but similar symptoms can respond to the same symptom-based treatment: (1) deep brain stimulation of the thalamus improves tremor symptoms in Parkinson’s disease and also essential tremor and (2) applying transcranial magnetic stimulation (TMS) to the dorsal lateral prefrontal cortex improves depression, independent of whether the depression is due to Parkinson’s disease or whether it represents a primary psychiatric illness [33]. This interventional approach is also consistent with a growing movement towards studying neuropsychiatric and neurodevelopmental disorders by focusing on specific symptoms, exemplified by the NIMH Research Domain Criteria (RDoC) initiative [31]. Applying this concept to individuals with ASD and other neurodevelopmental disorders would suggest that the observed heterogeneity in symptom burden across individuals may be due to the fact that each symptom is due to alteration of a different set of brain regions and networks. This could also explain the difficulty of identifying consistent findings across a group of individuals having a consistent diagnosis, but variable symptoms [15–17].

### Brain lesions provide stronger causal inference for symptom generation

Fortunately, there is a longstanding tradition of using causal information to understand brain function in humans, traditionally by studying the natural history of patients after brain injury. In contrast to the correlations produced by traditional neuroimaging, new-onset brain lesions can provide a *causal link* between a damaged brain location and a resulting symptom [35, 36]. Specifically, careful study of individual cases of focal brain injury provides a unique view into how specific behaviors and components of human intelligence are affected when components of the brain’s network are impaired or removed. A significant portion of what we know about human brain function has historically been based on these findings and is of particular importance in clinical neurology, e.g., expressive aphasia from injury to Broca’s area [35, 37]. This approach has also been applied to pediatric stroke and brain injury in attempts to predict developmental outcomes, e.g., after perinatal stroke [38–41] as well as to computational models to provide unique insight into the criticality of different brain regions [42, 43]. While ASD and other neurodevelopmental disorders are distributed (i.e., non-lesional) conditions, the study of brain lesions that result in specific symptoms, e.g., post-stroke agitation and aggression [44], that are also seen in individuals with non-lesional conditions, represent a valuable strategy for localizing symptoms; such studies are advocated by several groups [45–48], and increasing evidence suggests that consistent brain regions and networks are involved across conditions [31, 49].

### “Lesion network mapping” identifies brain networks that underlie complex lesion-induced symptoms

While examining lesion locations in individual patients provides significant insight, there are many reported cases where injury to multiple, distinct regions of the brain cause similar phenotypes, as well as the more common case that injury to singular regions of the brain produce a variety of disparate alterations in brain function, even in the same individual [50, 51]. It is now recognized that many brain functions (and thus many symptoms) do not localize to a single brain region, but rather depend on a circuit of connected areas [52]: a lesion to one of several regions within a circuit would lead to similar symptoms. While recent work has identified that lesions affecting “cortical hubs,” such as the default mode network, can produce widespread deficits, lesions affecting specialized networks are likely to produce deficits affecting a particular cognitive domain or function [53]. Extending this model further, it may be possible to identify specific brain circuits that are critical for the generation of specific symptoms by studying large collections of lesions with associated behavioral data; similar to how identifying multiple families with a consistent and specific phenotype can suggest a specific genetic alteration affecting a specific cellular pathway.

One computational neuroimaging method to interrogate the relationships between brain networks and phenotypes that is quickly gaining traction is “lesion network mapping” [50, 54, 55]. This method starts with a retrospective collection of patients with focal brain alterations, e.g., strokes, tumor resections, atrophy patterns, or neuromodulation, that are temporally associated with a particular change in behavioral phenotype. While it is difficult to prove causation in any single patient, the underlying assumption of this method is that if a brain network is consistently altered in affected patients with a consistent phenotype, or if the degree of network alteration correlates with the degree of behavioral phenotype, alterations of this network likely represent an endophenotype and should be considered more “causally” associated with symptom generation than any particular single lesion location. Evidence already suggests that such findings are relevant in non-lesioned patients as well [56].

The collated locations of brain alteration from each patient are then used as seeds for analyzing resting-state functional connectivity to understand which brain networks are affected by each focal lesion. While using post-injury resting state fMRI data from the patients themselves is useful in predicting outcomes, it is rarely available for most cohorts [57]. Instead, lesion network mapping leverages the availability of large-scale collections of resting state fMRI data from normative/typically developing participants [55]. This answers the question: “what brain network would be impacted by a lesion prior to any chronic compensation or plasticity?” This is analogous to comparing a patient’s specific mutation to a normative genome. Examples of these “normative connectomes” include using 1000 healthy participants from the Brain Genomics Superstruct Project (GSP) with an average age 21.5 ± 2.9 years [58] or 1000 typically developing participants from the Adolescent Brain Cognitive Development (ABCD) with an average age 9.2 ± 0.2 years [23]. The collection of normative network maps from each lesion can then be statistically compared to each person’s behavioral measures to identify which connections, when impaired, are consistently associated with symptom severity in a sensitive and specific way [54].

An additional benefit is that since only the structural MRI data are needed from patients to identify lesion locations, this technique is applicable to the wide range of neurodevelopmental and neuropsychiatric symptoms that can occur after brain injury. In fact, lesion network mapping has identified brain circuits for a number of distinct—and often complex—neuropsychiatric symptoms [54]. For instance, recent work leveraging data from 713 patients across 14 datasets found that a consistent and specific brain network, including the intraparietal sulcus, dorsolateral prefrontal cortex, inferior frontal gyrus, ventromedial prefrontal cortex, and subgenual cingulate cortex, was affected in patients with depression, i.e., lesions connected to this network were associated with increased depression, while TMS and DBS sites connected to this network were associated with improvement in depressive symptoms in patients *without* brain injury [59]. Other work has found specific network alterations associated with mania [60], hallucinations [61], and criminal behavior [62], as well as more focused neurological symptoms such as blindsight [63], freezing of gait [64], and cervical dystonia [56] among others. Finally, lesion network mapping has also been adapted to make sense of traditional neuroimaging findings where a large collection of related studies does not seem to converge well, e.g., meta-analyses of brain locations involved in migraine revealed multiple disparate and seemingly unrelated brain locations, but a *coordinate network mapping* approach of this same data identified that these brain locations were in fact part of a singular network defined by connectivity to extrastriate visual cortex [65].

### Using lesion network mapping to identify networks that may be important in non-lesional neurodevelopmental disorders

This methodology is also particularly useful for choosing among multiple hypotheses where correlational approaches do not converge on a consensus neuroanatomical structure. One example of this is face recognition impairment [66]. As many as 40% of individuals with ASD have impaired or altered face-processing ability [67, 68] which significantly affects the development of social skills [69]. However, traditional correlational neuroimaging investigations studying participants with ASD have provided variable and contradictory results regarding which brain region or network is responsible for this difficulty [67, 70–72]. Since face recognition impairment can *also* be caused by focal brain injury, we applied lesion network mapping to identify brain circuits that, when disrupted, cause sudden-onset face recognition difficulty, i.e., acquired prosopagnosia [66]. This identified a circuit of brain regions positively connected to the right fusiform face area (FFA) [73] and negatively connected, i.e., have an inverse history of co-activation, to several regions of the left frontoparietal control network that are implicated in cognitive control, hierarchical task-set manipulation, and recognition of ambiguous visual stimuli [74–77]. Injury to these circuits also predicted subclinical facial agnosia in an independent dataset [66]. These regions are among a larger set of regions that have variably been reported in fMRI studies of ASD; however, the stronger linkage between damage to this particular network and specific loss of face recognition support a specific testable hypothesis. If studies of idiopathic ASD identify a relationship between impaired face processing and alterations of this same neuroanatomical circuit—which are currently underway—converging evidence would then suggest that this altered network represents an endophenotype of face processing in ASD and not simply a compensatory or correlational finding.

### Focal cortical malformations and tuberous sclerosis complex may provide an opportunity for direct lesion network mapping of neurodevelopmental disorders

An important caveat of using acute-onset lesions, often in adults, for lesion network mapping is whether the identified circuits are also important for neurodevelopmental disorders, which are typically not associated with a history of stroke, nor with obvious neuroanatomical abnormalities. However, lesion network mapping *has* proven effective for postoperative cerebellar cognitive affective syndrome, one of the few specific post-stroke syndromes that primarily affect children [78]. To bridge this gap, identifying pediatric clinical cohorts with more subtle focal cortical alterations may be informative. One such cohort is children with tuberous sclerosis complex (TSC), which is characterized by focal cortical tubers that affect the function of the surrounding cortex and a high risk for developing ASD [79–81].

As one example of how lesion network mapping may provide insight into neurodevelopmental symptoms, we recently found that connectivity between cortical tubers and the subcortical globi pallidi is a strong predictor of infantile spasms, a sudden-onset epileptic syndrome that affects up to 55% of children with TSC and is strongly associated with poor neurological outcome if not treated rapidly [82–85]. This study sought to predict an epilepsy syndrome and did not directly focus on developmental outcomes. However, this finding suggests that the location and connectivity of cortical tubers and other focal neurodevelopmental anomalies may *also* be useful in identifying other brain networks critical for symptom generation in children with TSC. While there is reason to suspect that the neural abnormalities in TSC are not restricted solely to tuber locations [86, 87], prior studies have already suggested potential relationships between tuber location and neurodevelopmental outcomes, albeit with small sample sizes and non-computational, e.g., visual inspection/counting, approaches [88–93]. Moving forward, we have studies currently underway determining the relationship between tuber location and connectivity with face recognition ability and ASD-related symptoms in TSC using lesion network mapping and complementary approaches.

### New neuromodulation methods allow for non-invasive direct testing of neuroanatomical hypotheses

New therapeutic approaches, such as non-invasive transcranial magnetic stimulation (TMS) and transcranial direct stimulation (tDCS), enable targeted *exogenous* stimulation, or suppression, of specific brain circuits, and have become an established technique for studying cognitive processes [94–96]. One of the attractions of non-invasive neuromodulation is that it fills an important methodological gap in our ability to study human cognition. Namely, while neuroimaging and neurophysiological techniques can identify changes in brain activity or connectivity correlated with a cognitive task or behavioral phenotype, they lack causal inference, as discussed above. Similarly, while lesion studies provide causal inference for the involvement of particular tasks in a cognitive task or behavioral phenotype, finding cases of damage to all regions of interest can be difficult and the extent and the mechanism of the lesion is not always consistent. TMS, tDCS, and other focused neuromodulation techniques sidestep these two limitations by allowing for direct manipulation of neural activity in any research participant with a reasonable spatial and temporal resolution [97].

Given these capabilities, TMS has been leveraged over the past two decades to explore a variety of cognitive processes including attention [98], learning [99], awareness [100], plasticity [101], language [102], and perception [103] where TMS to specific brain regions interrupts specific brain function, which can also be observed in real-time with fMRI [104]. TMS has also been explored specifically for biomarker development in ASD as it can measure intracortical inhibition, facilitation, and plasticity—all metrics found to be altered in models of ASD—through paired pulse and repetitive TMS paradigms. It has also proven useful in the study and treatment of numerous clinical applications, including movement disorders [105], epilepsy [106], Tourette syndrome [107], depression [108–110], obsessive compulsive disorder [111], schizophrenia [112], and the spectrum of generalized anxiety, posttraumatic stress, and bipolar disorders [113]. Trials are also already underway for TMS in autism, e.g., focusing on the right temporal-parietal junction (rTPJ) [114] and dorsolateral prefrontal cortex (DLPFC) [115] among others. However, as noted above, the evidence for “which” target to use is mixed and largely correlational and results thus far have been mixed.

Nonetheless, there are limitations to exogenous stimulation techniques that can make interpretation of these studies complicated. Specifically, while techniques such as TMS are often described as “injecting noise” into a particular region [96], or as creating a “virtual lesion” [95], the specific and complete effect of exogenous stimulation, or suppression, is still debated [116]. Similarly, it has also been proposed that some or all of the effect of focused neurostimulation may occur indirectly, through connections to a distributed network [117], a hypothesis that appears to be true in depression [110]. This has led to a rise in multi-location, “double-coil,” neuromodulation experiments, to assess how stimulation at one location modulates the activity of another [118, 119], and protocols that allow for the combination of TMS with positron emission tomography (PET), electroencephalography (EEG), or fMRI, to assess the network effects of neurostimulation in relatively real-time [120, 121], with some groups taking this further to combine TMS, fMRI, and EEG simultaneously [122].

From a clinical standpoint, while neurostimulation techniques have demonstrated clear therapeutic utility in a number of neuropsychiatric conditions; patients often have difficulty tolerating the stimulations, and their utility in patients with ASD, or indeed in pediatrics in general, may be limited [123–125] and developing alternative protocols or modalities for pediatric use is an active endeavor [126]. There may also be significant gains from taking advantage of the brain’s own neuromodulatory and mechanisms governing plasticity, and methods aiming towards *endogenous* modulation of the brain have been investigated for decades. While bio- and neurofeedback methods have traditionally focused on externally measured biological, or EEG-based, indicators that typically lack spatial specificity, recent technical advances now permit real-time monitoring and control of specific brain regions and networks via feedback that is obtained during fMRI [127–131]. However, even without spatial localization, neurofeedback paradigms may be effectively for specific symptoms in ASD, e.g., potentially improving components of executive function by reducing atypically heightened theta/beta ratios by inhibiting theta activation and enhancing beta activation [132].

### Real-time fMRI neurofeedback

Real-time functional magnetic resonance imaging neurofeedback (rt-fMRI NF) was first piloted in 1995 [133] and has since become a small but rapidly developing field. Like exogenous neuromodulation techniques, rt-fMRI NF can also be used to examine the relationship of neural activity and cognitive functions and, at the same time, also serve as a clinical tool to mitigate a host of clinical symptoms. Neurofeedback based on rt-fMRI works by providing a training protocol that allows participants to voluntarily control their brain activity and/or measures of connectivity [128, 130, 134–137] as measured by fMRI as the BOLD response from a targeted region of interest (ROI), network of regions, or a computed difference between regions. The neurofeedback loop is closed when this brain activity or calculated measure is presented as a feedback signal to the participant being scanned in near real-time. Given the time lagged nature of the BOLD response, closed-loop times typically range from 2 to 10 seconds or can be presented at the end of task blocks [130, 138]. With the help of neurofeedback, participants can learn voluntary control over the own brain activity and connectivity with a goal of transfer to experimental situations without feedback and to, hopefully, generate long-term changes in brain activity and behavior. In fact, initial testing of rt-fMRI NF in ASD, e.g., to upregulate FFA activity [139], superior temporal sulcus activity [140], or to modulate “aberrant” brain connectivity [141], has already begun. As one example, Pereira et al. recently demonstrated with adolescents with ASD, as well as typically developing adolescents, that 2 sessions of rt-fMRI NF was sufficient to upregulate bilateral FFA baseline activity, with participants with ASD showing a larger increase, but was not associated with behavioral improvement—likely due to the brief intervention and short time scale of this feasibility study [139].

In addition to its use as an investigative tool, effective clinical use of rt-fMRI has emerged for such conditions as chronic pain [142], addiction [143, 144], stroke recovery [145], Parkinson’s disease [146], tinnitus [147], autism [141], depression [148], psychopathy [149], and emotional face processing in schizophrenia [150]. Unlike several methods noted above, rt-fMRI NF requires no external stimulation and can be utilized in all patients that can tolerate a standard task-based fMRI protocol [131]. Conversely, rt-fMRI NF is not going to be useful for patients with profound autism and children with significant developmental delay who cannot participate in a task paradigm, nor even hold still for long periods of time in an MRI machine. As such, it may usefully serve as both an option for children with less severe cognitive and sensory profiles as an alternative to TMS and as a platform for rapid proof-of-principle investigations that would then lead to TMS/tDCS protocols. It can also confirm whether modulating a particular hypothetical target modulates the network of interest in brain structures that would normally require deep brain stimulation to reach. Simultaneous EEG acquisition in these patients may also identify a signature that correlates with successful fMRI-guided neurofeedback, unlocking the possibility of targeted neurofeedback in younger children who cannot participate in rt-fMRI NF [151, 152]. New developments in fMRI, such as the use of VR and hyperscanning (where two participants can interact while being scanned in two different MR scanners at the same time) allow for highly immersive and interactive paradigms that may prove increasingly tolerable for pediatric participants and allow for investigation of social skills [153–155].

## Conclusions

Over the past decade, significant efforts have been put into improving traditional cognitive neuroscience approaches to allow far more inference to be drawn from observational and correlational techniques. This has included the generation of large-scale datasets that are multiple orders of magnitude larger than previously available and a convergence with computational neuroscience approaches to better understand the heterogeneity inherent in these data. This is converging with a progressive standardization of experimental methods and a heightened level of “deep phenotyping” that allows for detection of small effect sizes in clinical populations. Now, the development of several paradigm-changing methods that with increased causal inference between alterations in brain structure and function with human behavior has the potential to provide not only new insight into typical brain development and neurodevelopmental disorders, but also an avenue for targeted therapy for specific symptoms, disabilities, and impairments.

I propose that significant progress can be made towards this goal by combining the techniques described here into an interactive pipeline of multimodal investigation. Specifically, (1) the generation of circuit-based hypotheses for individual symptoms and behaviors from clinical cohorts with lesions, tubers, tumor resections, and other focal brain alterations, (2) validation of these symptom localizations through prospective neuroimaging study of patients with neurodevelopmental disorders with similar symptoms and retrospective analysis of large-scale highly characterized cohorts, (3) testing whether these circuits can be modulated through non-invasive therapy, e.g., behavioral, rt-fMRI NF, or TMS/tDCS-based interventions, and (4) assessing whether non-invasive modulation of these circuits results in measurable changes at the group- and individual-level. This approach focuses directly on symptoms, cognitive processes, and behaviors in human participants and side-steps concerns regarding animal model face and construct validity [156], as well as the prolonged therapeutic pipeline in traditional bench-to-bedside translation [157]. As such, a paradigm of “bedside-to-bedside” translational research that identifies, validates, and assesses the efficacy of transdiagnostic treatment targets is both feasible and attractive for neurodevelopmental and neuropsychiatric disorders.

## Data Availability

Data sharing is not applicable to this article as no datasets were generated or analyzed during the current study.

## Abbreviations

ASD: Autism spectrum disorder
EEG: Electroencephalography
FFA: Fusiform face area
GWAS: Genome-wide association studies
PET: Positron emission tomography
RDoC: Research Domain Criteria
rt-fMRI NF: Real-time functional magnetic resonance imaging neurofeedback
tDCS: Transcranial direct current stimulation
TMS: Transcranial magnetic stimulation
TSC: Tuberous sclerosis complex
